# Challenged by patients: a qualitative study of clinical supervisions of endocrinologists conducted by psychiatric liaison clinicians

**DOI:** 10.1186/s12913-024-12030-8

**Published:** 2024-12-04

**Authors:** Nedjma Mazouni, Friedrich Stiefel, Céline Bourquin, Gundula Ludwig, Laurent Michaud

**Affiliations:** Psychiatric Liaison Service, Lausanne University Hospital and University of Lausanne, Avenue de Beaumont 23, Lausanne, 1011 Switzerland

**Keywords:** Supervision, Endocrinology, Liaison psychiatry

## Abstract

**Background:**

Clinical supervision by psychiatric liaison clinicians is frequently provided in medical settings such as oncology and palliative care, but rarely in endocrinology. Consequently, the specific psychosocial issues faced by endocrinologists in their daily clinical practice and how they deal with them remain largely unknown. We aimed to explore individual supervisions of endocrinologists to gain insight into what kind of clinical situations they present, how they react to them and how this is worked through in supervision.

**Methods:**

The data set consisted of eight audio-recorded first supervision sessions of endocrinologists conducted by liaison psychiatry clinicians, which were transformed into written core stories accounting for key components of each session. A secondary analysis of these core stories was conducted using an interpretative approach, focusing on (i) the types of clinical situations and (ii) the supervisees’ counter-attitudes towards patients. Additionally, particular attention was given to how the supervisors worked through these elements.

**Results:**

Endocrinologists presented patients who did not adhere to treatment, behaved inexplicably, or held moral values that differed from their own. Challenged by these situations, supervisees experienced negative emotions (e.g., anxiety, irritation, guilt), associated with behavioral reactions (e.g., avoidance) and/or defensive stances (e.g., denial, rationalization). In half of the supervisions, addressing these difficulties allowed supervisees to link key characteristics of the patient interaction with their own unresolved issues; in the other half, supervisees were less inclined to confront themselves with their own contributions to the patient interaction and the supervisor adopted a more active stance, making specific contributions (e.g. support, advise).

**Conclusions:**

The findings call for training programs addressing “difficult” patients and advocate for closer collaboration between endocrinologists and liaison psychiatry clinicians.

## Background

Clinical supervision in the psychiatric setting allows junior clinicians to present their interactions with patients to a supervisor, generally a senior colleague. Supervision aims to provide guidance and support to the supervisees by focusing on psychosocial and relational issues of the patient encounter (restorative function) and to improve their psychosocial, communicational, and relational competences (formative function), in the interest of patients’ welfare (normative function) [1–3]. Different beneficial effects of clinical supervision have been demonstrated: support, decreased feelings of loneliness, enhanced intergroup solidarity among supervisees, acquisition of psychological knowledge, and better understanding of patients, relational dynamics of the clinician-patient relationship or own reactions [4–6]. Moreover, supervision has been linked to the retention of workforces [7] and diminished burnout [8] and can thus be considered as a means to support the supporter [9].

Individual or group supervision of clinicians, first introduced in the field of mental health [10], are meanwhile present in various medical settings [11] especially in oncology and palliative care [1]. In these settings, the supervisor is usually a liaison psychologist or psychiatrist, who has experience and competence in the somatic context, be it with patients (consultation activity) or with health care professionals (liaison activity) [11]; for the interested reader, this last literature reference discusses clinical supervision in general and its definition as a formal process of professional support and learning enabling practitioners throughout their professional career to develop and update their knowledge and competences.

While the challenges of the patient encounter have been acknowledged over the past decades, they are often reduced to communicational difficulties. Consequently, communication training has been implemented in some medical settings. However, communication trainings have limited effects on patient outcome [12] and most often focus on technical aspects such as advice how to structure information or how to respond to emotions but neglect the relational dimensions of the patient encounter and clinicians’ own contribution to difficult interactions with patients [5]. We have therefore argued that these trainings should be complemented by more reflexive approaches, which address the patient-clinician relationship and the psychology of the clinician [13]. To address these issues, supervision is considered as a powerful means. When we mention the notion of “supervision,” we refer in the following to supervision by psychiatric liaison psychiatrists and psychologists, knowing that in the medical literature the term supervision or clinical supervision is used in a broader way and usually referring to the medical practice as a whole. The history of supervision in the psychiatric setting dates to the early psychoanalytic movement [14], and different supervisory models and techniques has been developed since then [15]. The main aim of supervision in the medical setting is to develop or increase the supervisees’ conceptual and clinical abilities to address the psychosocial dimensions of care and to set into motion a reflexive process, especially in situation when they feel “stuck” with a patient [16, 17].

While clinical supervision for mental health professionals has been the subject of numerous studies [2, 18], research on supervision in the medical setting is scarce and almost exclusively limited to the evaluation of its acceptability and self-reported impact [1, 19]. However, supervision can also be a valuable source to gain insight into psychosocial issues faced by clinicians working in a specific setting [20]. This is especially the case in settings, which do not have a long tradition of collaboration with liaison psychiatry [21] as it is the case of endocrinology. To the best of our knowledge, supervision is rarely implemented and has not yet been investigated in the endocrinology setting. This is the more surprising given the observation that physician-patient interactions, for example in diabetes [22–24] or obesity care [25], influence disease-related distress, adherence to treatment or medical outcome. Furthermore, affective disorders and psychosocial issues frequently accompany endocrine diseases, potentially impacting the patient’s quality of life [26, 27].

This study is a secondary analysis of stories constructed from supervision sessions between liaison psychiatrists/psychologists and endocrinologists, which were audio recorded as part of a communication training program. It aimed to gain insight into what kind of clinical situations endocrinologists bring into supervision, how they react to them and how this is worked through with supervisors.

## Materials and methods

 Our study followed a medical student master thesis [28], which was focused on the relationships at play in the supervision of endocrinology physicians. It is a secondary analysis of the core stories created as part the master thesis and accounting for key contents of supervisions. The material of the present study (Sect. 2.1) is the same as the master’s thesis, while the data analysis differed (Sect. 2.2).

### Material

#### Context of data collection

For the second time, residents, and chief residents of the Service of Endocrinology, Diabetology and Metabolism at Lausanne University Hospital benefited from a three-day communication training program in the year 2022. The training was conceptually adapted from the Swiss communication training for oncology clinicians, which was initiated in 1998 by the Swiss Cancer League and became mandatory for clinicians who specialize in oncology and hematology in Switzerland [29]. Ten endocrinology clinicians participated in the training, which was led by a psychiatrist and a psychologist, both with a long-standing experience in liaison psychiatry, communication training and supervision. The first part of the training, spanning two days, consisted of case discussions, role-plays and analyses of participant’s videotaped interviews with a simulated patient, as well as a short theoretical presentation on communicational and interactional aspects of the patient encounter. It was completed by three individual supervision sessions for each participant. Six months later, during a third training day, the issue of psychosocial distress and depression was addressed, based on role-plays among participants with a simulated patient and short theoretical inputs. Participating endocrinologists treat a variety of specific patient populations: some work exclusively with obese or diabetic patients, others with patients with various endocrinological disorders and occasionally patients requesting medical assistance for gender transition.

Supervision sessions were conducted by senior liaison psychiatry clinicians with extensive experience in supervision of colleagues working in somatic medicine. Supervisors are psychodynamic-oriented therapists. Consequently, they focused on the relationship between patients and supervisees, with the main objective to increase relational competence, self-reflection, and understanding of conscious and unconscious dimensions in clinical encounters [30, 31].

Participating endocrinologists were orally informed about the study, received an information sheet, and provided written consent. The consent form left open the possibility that the material will be used for different analyses.

#### Used data material

Of the ten potential participating endocrinologists (i.e., supervisees), eight were retained for the study, with their first supervision session included in the material. Two additional supervisions from other endocrinologists were excluded: one because the audio recording was lost, and the second because it was conducted by a third supervisor. We considered it important to maintain a certain homogeneity in the material by focusing on supervisions conducted by two supervisors, thus ensuring a sufficient volume of material from each to allow for meaningful analysis. The dataset for the master thesis thus included 8 first supervision sessions of endocrinologists lead by two co-authors of this article: FS (*n* = 5 supervisions) and GL (*n* = 3). Supervision sessions were audio-recorded and lasted between 45 and 60 min. Core stories were created on the basis of the audio recordings as part of the master thesis and served as data for the secondary analysis, carried out in the present study (section Data analysis). The master thesis [28] aimed to provide a thick description —a detailed and in-depth account— of the relationships at play in the supervisions [32, 33]. Several successive steps were taken, starting with the creation of comprehensive narrative reports that described the content of each supervision session, the dynamics of interaction between the patient and clinician, the issues raised by the supervisee and how the supervisor handled them. Subsequently, key contents and passages were identified, forming the basis for a *core story* for each supervision. They were distinguished by the space devoted to address a certain topic during supervision, the quantity of details reported on this topic, and the repetitive nature of discussion of this topic. However, we were also attentive to omissions, and to very brief reference to a subject, which may also have a core significance in supervision [34]. Key contents were reviewed and discussed among the research team. The approach followed the process of core story creation used in narrative analysis [35], which consists of the making of story-like structures through interactions between a supervisor and a supervisee in this situation.

#### Data analysis

The secondary analysis of the core stories was conducted using an interpretative approach, focusing on (i) the types of clinical situations and (ii) the supervisees’ counter-attitudes towards patients. Particular attention was also given to how the supervisors worked through these elements. Counter-attitudes are understood as reactions to patients that can manifest in various forms, such as emotions (expressed or not, e.g., anger), attitudes (e.g., avoidance), or behaviors (e.g., deviation from good medical practice). We explored these counter-attitudes by specifically examining the emotions, attitudes, and behaviors described in the eight core stories. We used an interpretive approach informed by psycho-dynamic theory. Psychodynamic theory is based on a few key assumptions, for example the view that early development during childhood may shape future experiences and construction of relationships, that non-conscious dimensions of our mind orient our ways of being in the world or that defense mechanisms, unconscious psychological responses protecting from painful feelings, mediate between inner needs and outer reality [36]. Psychodynamic approaches, primarily displayed in therapeutic settings, have also been used to analyze collectives, as exemplified by systems psychodynamic approaches to organization studies [37–39] and in health care organizations [40–42]. In previous investigations, we have used psychodynamic theory to analyze how physicians deal with their emotions [43] and counter-attitudes towards patients [44]. In our study, the two supervisors (FS and GL) were also investigators interpreting the material they co-produced with the supervisees. Consequently, they did not operate from a neutral stance. The supervisors’ interpretation and influence relate to their theoretical model and supervisor qualifications. First, they anchored in psychodynamic theory. Therefore, they focus on some aspects of the descriptions provided by the supervisees and leave out others. The desire to achieve goals such as enhancing supervisees’ insight or exploring their emotional resonance and counter-attitudes towards patients might influence the interpretation of results. For example, supervisors’ desire to improve the supervisees’ insight might influence the supervisors’ perception of supervisees’ moments of insight when analyzing the date. Second, the two supervisors are experienced liaison psychiatrists/psychologists capable of motivating supervisees and producing relevant material. Most importantly, they did not have hierarchical, professional, or private relationships with the supervisees (they did not consult in this setting), which could have an inhibitory effect on the supervisory process. From a methodological point of view, our proceeding aligns with the call to research supervision with experienced supervisors [1]. Finally, the two supervisors-investigators were part of a research team, discussing the results and reaching a consensus when necessary. The team consisted of three senior psychiatric liaison clinicians, with longstanding experience in supervision (FS, GL, and LM), a medical student (NM) and a social scientist (CB) with extensive experience in qualitative research, who has worked for over 15 years in the same service as the three psychiatric liaison clinicians. The second author (FS) led the secondary analysis of the core stories. His interpretations were then discussed with CB and LM in a first phase, and with NM and GL in a second phase.

## Results

Tables 1, 2, 3, 4, 5, 6, 7 and 8 (Supervisions 1–8, in the following referred to as S1-8) present the *core stories* of the eight supervision sessions (part of the master thesis’ results), which were the material for the content analysis of the present study. As detailed in Sect. Type of clinical situations, clinical situations reported by endocrinologists concerned mainly (6/8) patients, who did not meet supervisee expectations in terms of behavior (Patients who do not behave as expected) or moral values (Patients who do not share the same values as the supervisees). Section Counter-attitudes of the supervisees to the situations reports on the reactions of supervisees to the situations they felt challenged by: they were mainly emotional and behavioral, with various degrees of defensive states towards these emotions. Section Ways of addressing clinical situations and associated counter-attitudes presents how clinical situations and associated supervisee’s reactions were addressed in the supervision. For confidentiality reasons, elements of supervisees’ biographical material, provided in the core accounts of supervisions, were modified.

**Table 1:** The naturopath (S1)

The supervisee wishes to discuss two topics: non-compliance with follow-up consultations and a specific patient who is a naturopath and who links a symptom to one of the treatments (statins) prescribed by the supervisee, which he refuses to take. The supervisee suspects that the patient uses this symptom as a pretext to avoid taking the medication without telling his physician the real causes of this refusal. Regarding patients who miss follow-ups the supervisee reports that he shows understanding and tells patients not to worry about the missed consultation. Patients often state that they did not receive a message by text message recalling the appointment, an excuse which leaves the supervisee perplexed; however, the supervisee considers that patients are free to follow or not medical recommendations. Regarding the patient-naturopath, the supervisee observes that there are two categories of patients: those who do not complain and those who complain about the slightest symptom. For him, the naturopath clearly belongs to the second category. The supervisor first establishes a link between the two topics, both concerning the issue of non-compliance (the first by mean of passive resistance and the second by mean of active resistance). He then suggests that patients, who do not comply with follow-up consultations may have own beliefs regarding the necessity to be followed and adopt an attitude of avoidance. Regarding the naturopath patient, the supervisor thinks that he might be ambivalent towards traditional medicine. According to the supervisor, this ambivalence, as the non-compliance with follow-up consultations, should be addressed.	

**Table 2:** Stop the medication (S2)

The supervisee reports on a 60-year-old patient with diabetes, whose social situation has recently deteriorated due to the loss of his job. She feels irritation and frustration towards the patient since he does not comply with her recommendations regarding treatment and lifestyle. The patient indeed continues to take a medication she recommended to stop, because it provokes a mycosis, which could become severe. During supervision, the supervisee recognizes that she finds herself in a vicious cycle: she takes the time and energy to explain repeatedly and calmly to the patient why he should stop the medication, but he finally always ends up continuing to take it. Moreover, she considers that she should not show her irritation, for reasons of medical responsibility, but also because she believes that her continuous investment is beneficial for the therapeutic relationship she has with the patient. The supervisor suggests that the supervisee should consider how the loss of the job may have influenced the patient’s behaviour. He also highlights the contrast between the patient's experiential knowledge that the medication improves his diabetes, and that of the supervisee, who is aware of the risks of infection caused by this medication. The supervisor also brings up the idea of a certain ambivalence on the part of the patient, who knows that snacking worsens his diabetes, but probably finds some comfort in food (following the loss of his job). He then observes that patient adopts rather an attitude of waiting to be treated, than being engaged in his medical care. He then addresses the supervisee’s feelings of irritation and advises her to address the issue of non-compliance with the patient, with the goal that the patient positions himself and expresses his experience with medical care. The supervisor suggests several times that the medical authority of the supervisee is challenged by this situation, but the supervisee does not consider this hypothesis.	

**Table 3:** An unpleasant consultation (S3)

The supervisee wishes to discuss a patient with whom the consultations are “unpleasant”. The patient suffers from a cardiac myxoma and was fortuitously diagnosed with a neuroendocrine asymptomatic tumor, which needs surveillance. During consultations, the patient behaves aggressively and states that he doesn’t understand why he needs medical follow-ups, underlining that he has no desire to consult or have regular assessments. The supervisee reports that she gives a lot of importance to the relationship with patients and the associated alliance. The situation with this patient has thus questioned herself. She admits that she would like to terminate this follow-up and that she has adopted an avoidant behaviour with this patient. The supervisor starts to reassure the supervisee by stating that she made the best she could out of the situation and then observes that she had a lot to endure with this patient. He invites her to express her feelings and addresses the supervisee’s avoidant attitude, apprehending consultations, which she validates. He considers that the supervisee has had to "put up with the patient's constant discontent”, without being able to act. He concludes that the situation is double difficult, not only because the patient doesn't want to be treated, but also because he reacts aggressively. The supervisor establishes a link between the patient’s and the supervisee’s behaviour by pointing out that both show an avoidant attitude, the patient avoiding a confrontation with the disease and the supervisee avoiding an aggressive and non-compliant patient. After having explored the feelings of calling as part of the professional identity of the supervisee and the relational patterns she develops in general, the supervisor learns that she often occupies the role of a mediator, placing herself outside the conflicts, a general attitude, which undermines her position in this situation.	

**Table 4:** The mother (S4)

The supervisee reports on a female patient from Sri-Lanka, who is affected by diabetes of an auto-immune origin. The management of the diabetes is difficult since eating seems to be one of the patient’s only remaining pleasures. The supervisee receives by weekly text messages glycaemic results from the nurse who visits the patient at home, which makes him feel desperate. He doesn’t know what to do anymore with this patient, who takes the medication, but doesn’t change her eating habits, despite medical advice. Regarding his personal engagement, the supervisee considers that it is rather unusual for him to be engaged to such a degree. While he considers that it is important to be engaged, he also thinks it is important to maintain a certain distance, which is not the case anymore in this situation. The text message he receives reminds him regularly how the patient puts him in a situation of defeat provoking feelings of guilt. Guided by the supervisor’s questions, the supervisee acknowledges that the patient reminds him of his mother. He indeed had to help his allophonic and sick mother during her divorce by translating for her during legal and medical consultations. The supervisor shares his own experiences with cultural differences. Finally, the supervisor attempts to alleviate the supervisee’s guilt and concludes that the patient reminding him of his sick mother might add to the feelings of guilt he experiences.	

**Table 5:** Trans-identity (S5)

The supervisee wishes to talk about a patient he qualifies as “particular” admitting not having encountered this kind of patients prior to entering the field of endocrinology: transgender patients. He feels constraint to treat these patients, while his religious background does not approve such requests. The supervisee reports that he copes with the situation by evacuating the “non-medical part” of himself (splitting). However, he still maintains a somehow rigid attitude towards transgender patients and underlines that he generally values the relationship with patients and that he regrets not being able to create more solid ties with transgender patients. After validating that the medical care of transgender patients might be complex, the supervisor tries to explore the supervisee’s emotions, which are discomfort and annoyance in the presence of these patients. The supervisor underlines that splitting of parts of oneself can become problematic in a profession where human aspects occupy an important place. The supervision terminates by a discourse of the supervisor, which aims to provide some guidance of how to establish a relationship with a person who shows an otherness which is difficult to understand, illustrated by his own experience with patients.	

**Table 6:** The sisters (S6)

The supervisee brings a problem into the supervision regarding a “trait” he observed he has, and which bothers him in the way he conducts his consultations: his exaggerated need to assure himself that patients receive all the information and that they understand everything. The dilemma he faces is that he does not know for whom it is beneficial to receive all this information and who feels invaded by it. He also reports a difficulty to distinguish between information he provides for his own safety (in the legal sense) and information provided to protect the patient. The supervisee then justifies his attitude by examples he encounters of physicians in his entourage, who faced legal problems. He summarizes his thoughts by stating that his job is to care for patients and not to go to the court. He also evokes the idea that his attitude could be a compensation for not being enough present in the consultations, during which he is regularly disturbed (phone calls, etc.) and adds that he always had high exigencies towards himself, having been raised with two sisters suffering from handicaps and always preoccupied to relieve his parents. The supervisor underlines a parallel between his attitude as an educator teaching a pupil-patient, a comparison, which is recognized by the supervisee. He then tries to link the supervisee’s attitude with his position in the family, a proposition which is not endorsed by the supervisee. The supervisor finally suggests that the supervisee explores his emotions while trying to restrain himself to provide information and observes if he experiences anxiety.	

**Table 7:** Disrespect (S7)

The supervisee reports the situation of a 50-year-old patient with hyperthyroidism. The patient feels free to take medication as she wants and always desires to have the results of laboratory tests immediately; she often directly calls the supervisee to obtain these results. Finally, a conflict breaks out: on a day the supervisee already feels overwhelmed by his workload, the patient calls him directly to change an appointment, bypassing the endocrinology clinics’ desk. Irritated, the supervisee calls back the patient and asks her to excuse herself for having disturbed him. In the following, the patient decides to change the physician. The supervisee deplores that in certain cases a collaboration between patient and physician cannot take place. While he attributes this outcome to the patient’s personality, it still induces a feeling of failure, since the patient had compared him with her previous physician leaving him with the impression that he was not up to the task. In the following the supervisee distinguishes between being respected as a person and being recognized for the work he accomplishes. In the situation reported, he considered that the patient did not respect him as a person. The supervisor reassures the supervisee, underlining that the problem has been created for the most part by the patient’s personality and then tries to question the supervisee’s emotional (feeling of humiliation) and behavioural reaction (calling patient to demand an apology). Yet, any attempt to deepen the supervisee’s shaken self-esteem fails. The supervisor therefore explores contextual reasons (workload), which could have contributed to this conflict and the decision to call the patient.	

**Table 8:** Breastfeeding (S8)

The supervisee reports on a patient, who twice came with a delay, a fact which irritated her during the whole consultation, feeling guilty towards the other patients who are on time and must wait. The supervisee wishes to discuss this patient to learn how to keep a certain “neutrality” with this patient and to not distance herself. The supervisee then focuses on the bizarre behaviour of the patient, mentioning that she continues to breastfeed her 5-year-old son. While the patient denies this observation, the prolactin level being high, the supervisee suspects her to lie. Moreover, the supervisee questions if the patient is really a lawyer, as indicated. The supervision allows the supervisee to recognize that she negatively judges her patient and to add that not being able to stop breastfeeding makes her anxious (she has an 8-month-old baby). At the end of the supervision the clinician admits that her problem with the patient rather concerns the non-admitted and ongoing breastfeeding and not the initially mentioned delays.	

### Type of clinical situations

#### Patients who do not behave as expected

Supervisees tended to present situations in which the patient did not conform to their representation of the “good” or “normal” patient. In S1, the patient missed follow-up consultations and thus did not conform to his expected role of engaging in care; in S2, the patient failed to follow the physician’s advice on treatment (continued to take medication despite the doctor’s recommendation against it) and lifestyle; and in S3, the patient questioned the pertinence of the follow-up. This is also true for the patient in S7, who was intrusive with the physician by calling him repeatedly.

#### Patients who do not share the same values as the supervisees

In these situations, the patients had moral values, which were not shared by the supervisees. The divergence concerned trans-identity (S5) and the duration of breast-feeding (S8). Regarding the former, clinicians’ religious orientations interfered with the patient’s request for medically assisted transition to another gender. As for the latter, clinician’s own ideas about the duration of breast-feeding lead her to feel irritated, judging the patient.

### Counter-attitudes of the supervisees to the situations

The supervisees showed emotional and/or behavioral reactions (counter-attitudes) in the reported situations. Among the emotional reactions were feelings of impotence (S1), anxiety (S1, S2, S3), irritation, which remained contained (S2, S8), guilt (S4, S7), feeling constraint (S5) and regret with an experience of failure (S5, S7). Behavioral reactions included, in situations with anxiety (S1, S2, S3), avoidance by the supervisee, who did not dare to address the conflictual nature of the patient-physician relationship. One clinician, however, did not hold back his anger of feeling disrespected (S7) and firmly insisted on an apology from the patient after his repeated phone calls. Moreover, supervisees adopted different defensive maneuvers against painful emotions. In S1, the statement of the supervisee that patients are free to follow or not medical advice, might indicate feelings of impotence, which were dealt with by defensive rationalization. In S2, the supervisee reported a conflictual situation, which remained unaddressed by the parties involved; the associated feelings of irritation were contained and the repeated attacks on the physician’s authority denied. In S5, supervisee resorted to splitting to cope with his feelings of being constrained.

### Ways of addressing clinical situations and associated counter-attitudes

In half of supervisions, the working-through by supervisee and supervisor allowed to link the clinical situations to physicians’ own unresolved issues. In S3, anxiety was related to conflictual situations as the supervisee was used to deal with conflicts by adopting a mediator’s role in his private life. In S4, supervision allowed the supervisee to recognize that the patient reminded him of his mother and to understand that his guilt and attitudes of over-responsibility was related to past experiences and his position as only son in the family. In S6, the supervisee reported doubts concerning his way to inform patients (over-information), which might be guided by his own and not the patients’ needs. Supervision addressed how his attitude influences the clinician-patient relationship and a parallel was drawn between the doctor-patient relationship and the supervisee’s own relationship with a close relative suffering from a chronic illness. In S8, supervision allowed to understand how being also a mother with breastfeeding experience influenced the supervisee’s views on the clinical situation of a young mother. Of note, these four supervision sessions were conducted by the same supervisor. In the other supervisions, no specific links with supervisee’s own personal issues was made and the supervisor took an active position, illustrated by specific contributions. In S1 and S2, the supervisor advised the supervisee on how to cope with interpersonal issues with the patient; in S5, the supervisor somehow lectured about the issue of otherness; in S7, the supervisor primarily adopted a supportive role, acknowledging and contextualizing the complexity of the situation.

## Discussion

### Clinical situations presented in supervision

During supervision, endocrinologists presented patients who did not conform to the expected “good” or “normal patient”: patients who did not comply, were aggressive and disrespectful, did not follow doctor’s advice, did not trust allopathic medicine, and/or did not share moral values of the supervisees. This might not be pure accident. Indeed, each medical discipline can be considered to have its specific psychological challenges. In the oncology and palliative care setting, for example, supervisees often evoke feelings of impotence and sadness over loss or report situations where identification with patients is operant [9, 45, 46]. These observations are in line with the emotional resonance existential dimensions of severe illness provoke in clinicians [47]. We thus hypothesize that the clinic of endocrinology puts the topic of normativity into play. Indeed, an important part of the activity of endocrinologists is spent on efforts to measure, correct, normalize, and adjust bodily states. Does the central role of measurement, which might influence the diagnostic listening to subjective reports of patients, excessively color the endocrinologist’ daily work experience? Furthermore - and considering also how the material implicitly reflects the limited information endocrinologists have on the social background of their patients (e.g., living and work situation) – it might be that the focus on measures somehow diminishes the view on the person behind them? The ambition of “normalization” might trigger negative experiences in clinicians who find themselves also challenged by patients who deviate from the (psychosocial) norm. Indeed, adherence is a central issue in endocrinology, for example regarding diabetic patients [48] or those affected by hypothyroidism [49], often considered silent diseases that do not always encourage active cooperation from patients. However, reported situations were not only situations of non-adherence but included other forms of “deviances.” One could even argue that choosing endocrinology may be motivated by a special attraction for precision and order. We concede that this is a rather daring hypothesis, but research has shown that career choices may be shaped by personal experiences. For example, persons who choose to become physicians reported more physical illness in their families [50] and women who choose to become psychiatrists reported more adverse childhood experiences such as physical abuse, psychiatric hospitalization of their parents or death of a family member [51]. However, one has also to consider, that norms and attraction to precision is also a characteristic of other medical disciplines; therefore, our hypotheses might not be specific for endocrinology. Only a cross-sectional investigation of supervisions in different setting could answer this question.

### Supervisees counter-attitudes towards patients presented in supervision

Facing these challenging situations provoked emotional reactions in the physicians, which deviated from what they usually feel towards their patients. These emotions were not always recognized but manifested themselves, as for anxiety, by avoidant behavior. Feelings of irritation towards a vulnerable patient who needs help, were contained, or even suppressed, except for one supervisee, who after containing his anger for a long time, could not hold back anymore with the consequence that the patient decided to consult another physician. Difficulties with negative emotions are understandable. Most physicians choose their profession for pro-social motivations, being sensitive to patients’ needs and vulnerabilities [52]. To experience aggressive feelings towards a patient is on the opposite spectrum of what physicians wish to experience, but as Holmes stated in the British Medical Journal: “Have we not all sometimes felt bored and irritated by certain patients, longing for the consultation to end? … Have we never imagined the death of a certain patient and the relief that would bring, not just to them but to us, their impotent carers?” [53]. However, not all physicians can feel, think, and communicate as Holmes, since aggressive feelings towards patients often provoke anxiety or guilt and are thus evacuated or censured [46, 54]. Suppressing emotions, however, has consequences such as losing the insight they provide. For instance, feelings of anger often indicate experiences of being hurt, while anxiety can signal threat. Without this information, we lose orientation, as reported by supervisees (e.g., loss of the self in splitting, loss of relationship by avoiding the patient, deviation from good medical care). Censoring of emotions operates when clinicians equal feelings with words and deeds, which are distinct categories. Feelings - even aggressive feelings - are not only “allowed” to experience towards patients, but contain valuable information about our patients, ourselves, and the relationship we establish with patients. A non-compliant patient might provoke anger, especially when the physician considers such behavior as a personal insult; to feel the anger allows to think about non-compliance and to accept that in certain situations, we as physicians are limited and that this is not the fault of anybody and must be accepted. Moreover, guilt, regret or feeling constraint - as observed in our study - add to work pressure. Unsurprisingly some supervisees resort to defenses such as denial, splitting and rationalization to be able to continue to care for certain patients presented in the supervision. While this might work in the short run, defenses distort reality and do not prevent from distress [55].

For the reader who is interested in research and reflections on difficult patient encounter and associated physicians’ experiences and wishes to deepen his knowledge, we refer to the literature [9, 46, 56].

### Achievements during supervision and supervision style

Our results also show that, in half of the supervision sessions, endocrinologists and their supervisors joint work allowed to discover links between past and the present and the private and the professional. The psychosocial factors that led to the encountered challenges - in both the patient and the clinician - could be pinpointed. At the contrary, in the other half of supervision sessions, such links were not addressed, and the supervisor favored a “top-down” approach, adopting more cautious, supporting or teaching position. To help physicians identify their own reactions (both emotional and behavioral) is one of the main goals of supervision fulfilling the formative (promote psychosocial competence), restorative (support clinicians) and normative (good medical practice) functions of supervision. In psychotherapy, therapists tailor their interventions to the patient by situating them on a continuum from supportive (“recovering”) and interpretative (“uncovering”) interventions, depending on the level of defense of the patient [57]. Further research, including interviews with supervisors, might answer the question of whether the same continuum operates in supervision, with supervisors “uncovering” personal issues with certain supervisees while avoiding this with others who they feel are less inclined towards introspection. Such research could also address the supervisor’s “style,” which certainly has an influence of such aspects: our results showed that one supervisor conducted all sessions in which personal issues were addressed, while the other conducted three out of the four sessions with a more active attitude. Conducting research on supervision, we observed that supervisors do adapt to the supervisee, but they also stick to their style. For instance, some supervisors always address systemic aspects of the situation presented such as the impact of the institutional context (workload, competitive atmosphere, etc.), while others solely concentrate on the emotional experience of the supervisee. As observed in one of the described situations, a supervisee reacted reluctantly to a supervisor’s repeated attempt to link the difficulty the supervisee encountered with a patient to his own biographical elements. Such a confronting style may explain the reluctance of the supervisee, but it might also be a general defensive reaction of the supervisee towards encountering a psychiatrist and towards the very idea of supervision.

Regarding the restorative function of supervision, one can assume that some supervisees felt relieved, for example when feelings of guilt were addressed and questioned, or when irritation was validated as understandable reactions. While a formal evaluation of perceived support is lacking in our study, it has been demonstrated that supervision allowed to diminish negative emotions towards patients in oncology clinicians [19]. Regarding the normative function of supervision (quality of care), one might assume that advice to address the issue of non-compliance, for example, should enhance quality of care, but we do not have follow-up data, confirming that supervisees were able to integrate the supervisors’ suggestions in their clinical work.

### Roles and added value of liaison psychiatry clinicians

Finally, we would like to comment on the roles and added value of liaison psychiatrists and psychologists in a somatic setting. There is certainly an added value to change from a pure psychiatric consultation model, in which a patient is referred to a mental health specialist, who does not have any relationship, to the model of liaison psychiatry in which psychiatrists and psychologists are integrated in the medical team. To name a few advantages of the liaison model: liaison clinicians can enhance the sensitivity regarding psychosocial dimensions of illness, for example by accompanying physicians and nurses on their rounds, discuss indications for psychiatric consultations prior to referrals, provide advice regarding the stances to adopt with patients presenting psychiatric disorders, support clinicians after traumatic events, participate in continuous education with topics related to their competences or intervene on different levels of the hospital [9, 58, 59]. Regarding supervision, it is important that liaison clinicians who conduct supervision, work in another medical setting of the hospital, since sensitive issues and biographical elements of supervisees might emerge during the supervisory process, which then might complicate the professional relationship between supervisor and supervisee.

## Limitations of the study

This is a first exploratory study of supervision in the endocrinology setting, based on a rather small sample. Another limitation might be in the fact that two investigators were also supervisors; we have commented on how this might have had some influence on results. Recording the supervisions might have inhibited some supervisees; however, our observation was that supervisees seem to quickly forget the recording when starting to speak about a patient encounter, which interested or preoccupied them. Finally, we did not assess how supervisees perceived and evaluated the supervision, which might have provided some clues regarding some observations such as the finding that a supervisee was reluctant to the supervisor’s remarks.

## Conclusions

The study results illustrate that supervision in endocrinology - which is not yet widely implemented - provides insight into the endocrinology clinic and allows to address challenging situations and to uncover emotions, which are at times censured or contained by clinicians. Such avoidance can harm both clinicians and patients and may even lead to deviation from good medical care. Investigating supervision of endocrinologists also informs about the psychological issues endocrinologists face in their practice, be they related to the patients, themselves, or the interaction. This information could be considered in training on communication or in other interventions aiming to enhance reflexivity, psychosocial competence, and support of endocrinologists. Finally, investigating supervision is also a mean to demonstrate that endocrinologists could benefit, as do other medical disciplines, from the presence of liaison psychiatrists and psychologists, who not only can be consulted for psychosocially complex patients, but also for helping clinicians in their struggle with patients and themselves.

## Data Availability

The datasets used and/or analysed during the current study are available from the corresponding author on reasonable request.
